# The Influence of Oxytocin on Maternal Care in Lactating Dogs

**DOI:** 10.3390/ani11041130

**Published:** 2021-04-15

**Authors:** Asahi Ogi, Chiara Mariti, Federica Pirrone, Paolo Baragli, Angelo Gazzano

**Affiliations:** 1 Department of Veterinary Sciences, University of Pisa, 56124 Pisa, Italy; chiara.mariti@unipi.it (C.M.); paolo.baragli@unipi.it (P.B.); angelo.gazzano@unipi.it (A.G.); 2 Department of Veterinary Medicine, University of Milan, 20133 Milano, Italy; federica.pirrone@unimi.it

**Keywords:** oxytocin, saliva, dog, maternal care, behavior

## Abstract

**Simple Summary:**

The role of oxytocin in regulating uterine contractions and milk ejection has been largely outlined. However, its central functions in neuromodulating the onset and maintenance of maternal care in domestic dogs are still unclear. Since the mother–infant interactions have a severe impact in determining later behavior in mammals, this study intended to investigate the possible correlation between salivary oxytocin and maternal care in 25 lactating dogs. Only a negative correlation between salivary oxytocin and sniffing/poking behavior was found. Therefore, salivary oxytocin cannot be considered a strong predictive biomarker of the quantity of maternal care in dogs. Moreover, the percentage of male puppies showed a positive correlation with salivary oxytocin and lateral nursing, which suggests a sex-biased parental investment in this species. These findings can be considered the first piece of the oxytocin puzzle in regulating dog–pup interaction.

**Abstract:**

In recent years, many studies on the role of oxytocin (OXT) in the onset of parental care, regulation of social bonding, and modulation of the emotional state have been published. However, its possible regulation of maternal behavior in lactating dogs has not been investigated yet. For this reason, the present study aimed at assessing potential correlations between salivary oxytocin and maternal behavior in 25 lactating Labrador Retriever dogs. Salivary concentrations of OXT (sOXT) were unrelated to the amount of maternal care except for a weak negative correlation with sniffing/poking behavior. Moreover, sOXT was positively correlated with the percentage of male puppies. Sniffing/poking behavior, in turn, was positively correlated with the duration of time the mothers spent out of the whelping box, while the number of male puppies showed a positive correlation with lateral nursing, a position known to provide puppies the easiest access to the milk. Taken together, these results suggest that sOXT may not be strongly associated with maternal care dynamics but could be correlated with sex-biased parental investment in dogs.

## 1. Introduction

The scientific interest in oxytocin (OXT) has recently shifted from its peripheral to its central activity. The role of OXT is in fact twofold: On the one hand, this nonapeptide acts at a peripheral level as a hormone; on the other hand, it acts at a central level as a neuromodulator. Concerning the hormonal activity, OXT is secreted into the systemic blood circulation mainly in response to parturition and lactation. In combination with increased OXT receptor (OXTR) expression, this hormone stimulates myometrium contractility and regulates the release of prostaglandins during parturition [1]. In addition, circulating OXT stimulates the contraction of mammary gland myoepithelium and regulates the release of prolactin during lactation [2].

Centrally, OXT mainly determines social and maternal behavior in mammals [3]. Regards mothering, OXT is involved in stimulating the development of maternal behavior, as well as in promoting the bond between mother and offspring [4]. This has been shown in humans, in whom plasma and salivary OXT levels are associated with mother–infant bonding [5]. Moreover, a negative correlation between salivary oxytocin levels and maternity blues severity was found [6]. Similarly, a study conducted in 74 women suggested that plasma oxytocin could allow early identification of subjects at risk for postpartum depression [7].

The association between peripheral OXT and maternal care is not the only evidence of the interdependency of these two factors: If on the one hand the OXT influences maternal behavior, on the other hand maternal behavior would seem to influence the OXTR methylation [8] and expression in the brain of the infants [9]. Moreover, mother–infant bonding would seem to allow the offspring to cope better with stress even in adulthood [10].

Using an ethological approach, Quintana and Guastella reviewed OXT-based mechanism and argued that the role of this neuropeptide in regulating behavior is best characterized by an allostatic model [11]. The allostasis is defined as “the process of maintaining stability through change by anticipating future changes in environmental condition” [11]. According to this definition, OXT system would support the adjustment of physiological set-points promoting adaptation, reproduction, propagation and survival of the species [11].

In canine species, the correlation between OXT and behavior has been mainly investigated in three macro-areas [12]: Genetics studies on polymorphism [13] and methylation [14] of OXTR; studies on intranasal OXT administration [15]; and studies on the possible association between social behavior and peripheral OXT levels [16]. However, no scientific literature on the possible role of OXT in regulating canine maternal behavior has been found.

It is well known how the early experiences of pups have a severe impact in determining later behavior in domestic dogs [17,18]. However, the literature on the impact of mother–litter interactions is quite limited and contradictory. In a study conducted in young adult dogs, Bray and colleagues found that puppies who received higher amount of maternal care were less likely to succeed in their training program [19]. In a study conducted through an owner-filled questionnaire, Tiira and colleagues [20,21] found that shy and fearful dogs experienced poor quality of maternal care compared with non-fearful dogs. Moreover, Guardini et al. [22] found that the quantity of maternal care during the first three weeks of puppies’ life allows them to better cope with stressful situations when rearing occurs in laboratory, whereas in another study of Guardini et al. [22], the behavior of puppies reared in a home environment seems influenced by the quantity of maternal care in an different way, since the amount of maternal care was associated to an increased display of separation-related distress and orientation to an unfamiliar person [23]. Finally, Foyer and colleagues found, in a sample of military German Shepherds, that puppies raised by mothers providing more maternal care showed higher social and physical engagement and more aggression [24].

Despite the scientific literature on lactating dogs showing breed differences in retrieving behavior [25,26], time spent out of box [25], and overall trend of maternal behavior [23,27], the different links found between quantity, or quality, of maternal care and behavior of the offspring could have a significant impact in selecting dams and preventing possible behavioral disorder in dogs. Moreover, if peripheral OXT of the mothers is related to their care towards the puppies, it could be speculated that OXT could become a predictive biomarker of maternal care.

The present study aimed to investigate if the saliva concentration of oxytocin was associated with maternal care provided to the offspring in lactating dams.

## 2. Materials and Methods

### 2.1. Subjects

Twenty-five lactating Labrador Retriever dogs with high levels of socialization towards people were recruited. Twenty-three of them were raised by the same breeder and the two remaining dams were raised in a similar professional breeding context, with a comparable social and environmental stimulation. The coat color, age, number of parturitions, litter size, and percentage of male puppies (M%) were considered (Table 1) and then statistically analyzed.

The whelping boxes employed in this study were very similar in size and layout. All of them provided to the mother the possibility to leave the box, preventing to the puppies the possibility to follow her. Each box was placed in a quiet whelping room without the presence of other dogs or any uncontrolled social contact.

### 2.2. Sample Collection

Saliva samples were collected by the breeders or a research assistant familiar to the dogs, previously instructed by a veterinarian expert in behavioral medicine. Saliva was collected from the mothers every three days, from day 3 of lactation until day 21, between 8 and 9 a.m.—right after the first 15-min walk out of the whelping room and just before the reunion with puppies. Salivette^®^ swabs (Sarstedt, Rommelsdorft, Germany) were used for collecting the samples. The swabs were gently put under the tongue and in the cheek pouches of the dogs for 60 s. Salivary OXT (sOXT) concentrations were measured using a Cayman Chemical ELISA Kit^®^ (Item #500440) (Ann Arbor, MI, USA) previously validated in dogs [28]. All samples were immediately refrigerated and brought to the ETOVET laboratory at the University of Pisa (Italy) for centrifugation and stocking at −20 °C, until they were analyzed in accordance with the manufacturer’s instructions. 

After each saliva sample, the litters were videotaped for 15 min, as soon as the dams were back from their walk. Videos were used for the analysis of maternal behavior, performed by an experienced observer blind to the sOXT concentrations. The video camera (a Sony^®^ HDR-CX190E by Sony Corporation, Kōnan, Minato, Tokyo, Japan) was positioned on a 150-cm-tall tripod located frontally to the whelping box. Each video was analyzed through a continuous sampling method with BORIS^®^ v. 7.8 [29], following a specific ethogram of maternal behavior (Table 2). The relative duration (percentage on total time) of each observed behavior was measured and then statistically investigated. To better understand the relevance of nursing behavior, a relative duration of total nursing was calculated by adding the duration of nursing lateral, ventral, and vertical (Table 2). Moreover, to measure the observational consistency and repeatability, a second experienced observer analyzed the 12% of videos for calculating inter-rater reliability through the Cohen’s kappa coefficient (κ).

### 2.3. Statistical Analysis

The data were analyzed through parametric and nonparametric statistical models, depending on their distribution, with IBM SPSS^®^ v. 25 (Armonk, NY, USA). Multiple comparison analysis of variance (ANOVA; *p* > 0.05) and then the least significant difference (LSD) test between percentage of male puppies (M%) in the litter and sOXT concentrations was performed. Additional statistical analysis was carried out with Spearman’s rank correlation coefficient (*ϱ*) between the quantitative assessment of maternal behavior and both sOXT concentrations and M% with a significance at 0.05 level. Regarding the trend of OXT over time, the ANOVA (*p* < 0.05) Huynh–Feldt correction for violations of sphericity was applied. Finally, the Friedman test (*p* < 0.05) was used to compare the mean duration of maternal behaviors over time.

The study received a favorable recommendation from the ethics committee (OPBA, Organismo Preposto per il Benessere Animale) of the University of Pisa, Italy (Decision #64/2018) in accordance with Directive 2010/63/EU.

## 3. Results

The agreement between the two observers, calculated on the 12% of the videos, was found to be excellent (κ > 0.9); therefore, only a single observer video analysis was statistically evaluated.

**Figure 1:**
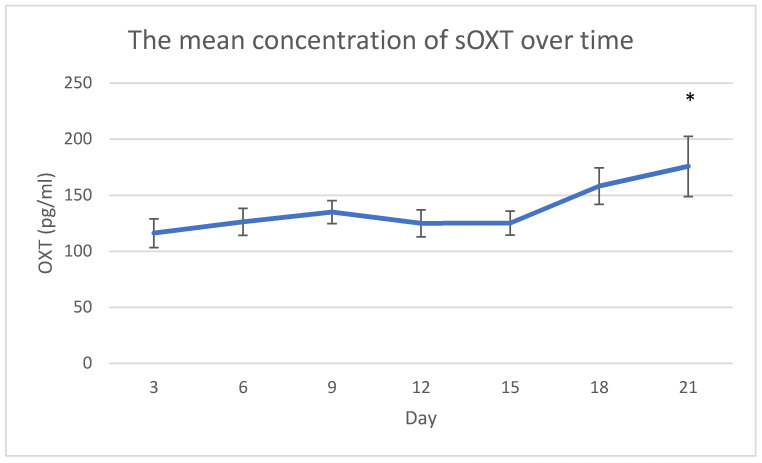
The mean concentration of salivary oxytocin (sOXT) across observations. Error bars indicate standard error of the mean. * *p* < 0.05 versus day 3, 6, 12 and 15.

The mean concentration of sOXT at day 21 was statistically higher than the mean concentration of sOXT at days 3 (*p* = 0.011), 6 (*p* = 0.031), 12 (*p* = 0.023) and 15 (*p* = 0.035). In general, the mean concentration of sOXT tended to increase over time, except for a slightly decrease at days 12 and 15 (Figure 1).

The statistical analysis showed no correlation between mother-related factors (reported in Table 1) and sOXT. On the contrary, analyzing the M% and sOXT with the LSD test, significant positive correlations were found (Table 3).

No significant correlation was found between the relative duration of any observed maternal behaviors and sOXT, except for a weak negative correlation with sniffing/poking (*ϱ* = −0.178). Moreover, no significant correlations were found between mother-related factors (see Table 1) and observed behaviors. The M% was positively, though weakly, correlated with the relative duration of lateral nursing (*ϱ* = 0.153) and retrieving (*ϱ* = 0.177) (Table 4).

Analyzing the relative duration of the observed maternal behaviors (for all correlations see Table 5), it was found that sniffing/poking behavior was positively correlated with the time spent out of box (*ϱ* = 0.228) and negatively correlated with total amount of nursing (*ϱ* = −0.228).

**Figure 2:**
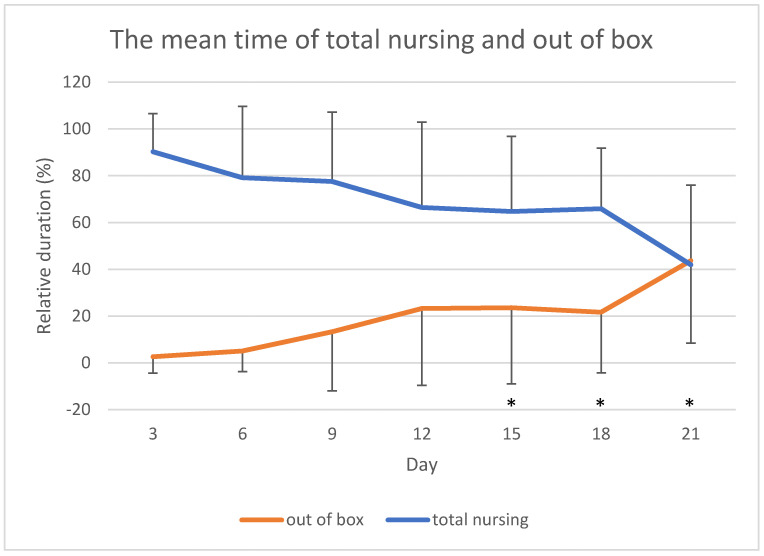
The mean time of total nursing (nursing lateral + nursing ventral + nursing vertical) and the mean time spent by the mothers out of box across observations. Error bars indicate standard deviation. * *p* < 0.05 versus days 3, 6 and 9.

Finally, the mean relative duration of time spent out of box by the mothers tended to increase during lactation period and, inversely, the total amount of nursing tended to decrease with time (Figure 2). In particular, the time spent out of box at days 21, 18, and 15 was significantly higher than at days 3 (*p* < 0.001 day 3 versus days 21, 18, and 15), 6 (*p* < 0.001 day 6 versus day 21; *p* = 0.011 day 6 versus day 18; *p* = 0.001 day 6 versus day 15), and 9 (*p* = 0.025 day 9 versus day 21; *p* = 0.022 day 9 versus day 18; *p* = 0.012 day 9 versus day 15).

## 4. Discussion

According to Bray et al. [19], who failed to find a positive correlation between quantity of maternal care and performance of the offspring in their training program, it could be assumed that an intermediate amount of maternal care can have positive effects on offspring resilience [19]. Moreover, many papers showed a correlation between anxiety of primiparous mothers and the amount of maternal care in mammals [30,31,32], including in dogs [33].

Consistent with this view, we can explain the negative correlation between sOXT and the quantity of sniffing/poking (*ϱ* = −0.178). This behavior, in fact, was positively correlated with the time spent out of box (*ϱ* = 0.228) and therefore negatively correlated with total amount of nursing (*ϱ* = −0.228). Higher amounts of sniffing/poking could be representative of higher amounts of time spent out of the whelping box and could suggest that dams with lower sOXT levels provide worse maternal care than dams with higher sOXT levels. Accordingly, excessive sniffing/poking behavior could be a sign of distress associated with lactation. A stress assessment of the mothers could have helped clarify this topic.

In contrast with the literature on dogs maternal behavior [25], in which only a temporary decrease in the amount of maternal care was described, we did not find a re-increase in the amount of maternal care after day 15. According to the text, the pups, starting at the second week of age, are able to proactively catch and “force” their mother to give them attention, thus increasing the total amount of maternal care. However, the whelping boxes employed in the present study did not allow the puppies to move outside freely, enabling the dams to move away from their puppies and gradually decrease the amount of their care over time.

In murine models, significant differences on the basis of pups’ gender in terms of received cares from the mothers have been reported [34,35], but very limited information on this topic has been reported in dogs. Dunbar et al. [36], in a study conducted on 32 beagle puppies, showed that the mothers have a first contact with the male pups in the 59% of the cases. This sex preference might be explained by behavioral response to olfactory stimuli. In rodents, dodecyl propionate, a compound released by pup’s preputial glands, which has been identified as an olfactory cue that helps mothers to identify pups that need to be licked, is higher in male pups than in female ones [37].

Despite litter sex ratio having been identified in previous studies in dogs as potentially affecting maternal care, no significant effect of the male/female ratio has been found [24,27,33]. Conversely, in the present study, the M% was found to be positively correlated with the concentration of sOXT and with lateral nursing (*ϱ* = 0.153), a position that provides puppies the easiest access to the milk [19]. Looking at this data from an evolutionary point of view, it could be assumed that the mothers spend more attention towards male puppies because it is more convenient in terms of fitness [38]. The M% was also found to be positively correlated with retrieving (*ϱ* = 0.177). However, this behavior was expressed only by three subjects and the data should be taken into account with caution.

Central and peripheral OXT did not show significant diurnal fluctuations in humans [39]. However, in our study, saliva and video samples were collected in the morning, at about the same time each day, in order to prevent a possible bias resulting from the sensory stimulation in the daytime routine of the dogs. Moreover, the correlation between central and sOXT should be higher in the early morning [40].

Measuring peripheral OXT in saliva has a variety of advantages. Firstly, collecting saliva is a non-invasive and low-stress sampling method. Secondly, measuring of sOXT concentrations offers the methodological advantage of not requiring a solid phase extraction [28]. Thirdly, sOXT seems to mirror central levels of oxytocin better than plasma OXT [40]. Finally, the half-life of OXT in blood is short, lower than 4 min [41], and the nature of lactation-induced release of OXT in blood is pulsatile [42,43]. On the contrary, despite the half-life of sOXT still being unknown, the most likely hypothesis is that OXT half-life in saliva is longer than OXT half-life in plasma [44,45].

## 5. Conclusions

This study found that salivary concentrations of OXT were unrelated to the amount of maternal care, except for a weak negative correlation with sniffing/poking behavior; therefore, sOXT cannot be considered a strong predictive biomarker of the quantity of maternal care in dogs. The correlation between M% and sOXT, as well as the correlation between M% and lateral nursing, suggests sex-biased parental investment in dogs. Further studies assessing the level of stress, personality, and oxytocin receptor gene polymorphisms of the mothers are needed to clarify the possible role of oxytocinergic system in selecting dams.

## Figures and Tables

**Table 1:** Subjects. The coat color, age, number of parturitions, litter size, and number of and percentage of male puppies (M%) of the 25 dams.

Subjects						
DOG	COAT	AGE (months)	PARTURITION (n°)	PUPPIES (n°)	M (n°)	M%
1	Yellow	43	2	5	3	60.0
2	Yellow	58	5	12	7	58.3
3	Black	41	2	5	0	0.0
4	Chocolate	23	1	9	8	88.9
5	Yellow	63	3	9	1	11.1
6	Black	81	3	4	1	25.0
7	Yellow	43	2	10	7	70.0
8	Yellow	38	2	8	4	50.0
9	Black	70	4	3	3	100.0
10	Chocolate	28	1	7	5	71.4
11	Black	30	1	8	3	37.5
12	Yellow	76	2	7	1	14.3
13	Black	59	3	9	2	22.2
14	Chocolate	50	3	6	5	83.3
15	Chocolate	38	1	6	4	66.7
16	Yellow	98	3	5	3	60.0
17	Black	79	4	6	2	33.3
18	Yellow	60	2	9	5	60.0
19	Chocolate	56	3	9	6	66.7
20	Chocolate	57	3	9	6	66.7
21	Black	66	4	6	3	50.0
22	Yellow	24	1	9	5	50.0
23	Yellow	34	1	8	5	62.5
24	Yellow	24	1	8	4	50.0
25	Yellow	94	4	9	2	22.2
Mean ± SD		53.32 ± 21.68	2.44 ± 1.19	7.44 ± 2.10	3.80 ± 2.10	51.20 ± 25.17

**Table 2:** Ethogram. The catalogue of maternal behaviors observed.

Ethogram		
Behavior	Definition	References
Out of box	The mother had her legs out the whelping box not providing maternal cares	Current paper
Contact	The mother was lying in the whelping box with elbows on the ground and in physical contact (tail and limbs excluded) with at least one pup	[24]
Licking	The mother was licking at least one pup	Modified from [24]
Sniffing/poking	The mother was sniffing, poking, or moving at least one pup around with the nose	Modified from [24]
Retrieving	The mother was carefully carrying in her jaws at least one pup	Modified from [26]
Nursing lateral	The mother was nursing (at least one pup suckling) while lying on her side or back, so that part, or all, of her nipples were exposed	Modified from [27]
Nursing ventral	The mother was nursing (at least one pup suckling) while lying on her stomach, so that her nipples were not easily exposed to the puppies	Modified from [27]
Nursing vertical	The mother was nursing (at least one pup suckling) while standing or sitting in the whelping box	Modified from [27]
Total nursing	Nursing lateral + nursing ventral + nursing vertical; nursing positions are mutually exclusive	Modified from [24]
Other	Any activity not assessable or not included in the behavioral catalogue	

**Table 3:** Least significant difference (LSD) test between the percentage of male puppies (M%) and salivary oxytocin (sOXT).

LSD Test between M% and sOXT		
M%	Mean Difference	Standard Error
11.1	−0.09	0.10
14.3	−0.07	0.11
22.2	−0.21 *	0.09
25.0	−0.39 *	0.10
33.3	−0.15	0.10
37.5	−0.18	0.10
50.0	−0.30 *	0.08
58.3	−0.22 *	0.10
60.0	−0.12	0.08
62.5	−0.43 *	0.10
66.7	−0.22 *	0.08
70.0	−0.30 *	0.10
71.4	−0.05	0.10
83.3	−0.52	0.10
88.9	−0.39 *	0.10
100.0	−0.22 *	0.10

* The mean difference is significant at the 0.05 level.

**Table 4:** The Spearman correlation coefficient (*ϱ*) between the percentage of male puppies (M%), sOXT and duration of the observed maternal behaviors: Contact, licking, total nursing (N_TOT) [nursing lateral (N_LAT) + nursing ventral (N_VEN) + nursing vertical (N_VER)], retrieving, sniffing/poking (S/P), and out of box.

The Spearman Correlation Coefficient (*ϱ*) between M%, sOXT and Maternal Behaviors									
	CONTACT	LICKING	N_TOT	N_LAT	N_VEN	N_VER	OUT OF BOX	RETRIEVING	S/P
M%	0.041	−0.109	−0.046	0.153 *	0.012	−0.075	−0.1	0.177 *	−0.085
sOXT	0.036	−0.093	−0.050	−0.078	−0.021	−0.056	0.020	−0.112	−0.178 *

* Correlation is significant at the 0.05 level (2-tailed).

**Table 5:** The Spearman correlation coefficient (*ϱ*) between the duration of the observed maternal behaviors: Contact, licking, total nursing (N_TOT) [nursing lateral (N_LAT) + nursing ventral (N_VEN) + nursing vertical (N_VER)], retrieving, sniffing/poking (S/P), and out of box.

The Spearman Correlation Coefficient (*ϱ*) of Maternal Behaviors									
	CONTACT	LICKING	N_TOT	N_LAT	N_VEN	N_VER	OUT OF BOX	RETRIEVING	S/P
CONTACT	1.000	0.064	−0.246 **	0.132	0.349 **	−0.220 **	0.018	−0.096	0.072
LICKING	0.064	1.000	−0.023	0.027	0.272 **	−0.013	0.087	0.020	0.557 **
N_TOT	−0.246 **	−0.023	1.000	0.165 *	0.107	0.320 **	−0.526 **	−0.072	−0.187 *
N_LAT	0.132	0.027	0.165 *	1.000	0.357 **	−0.549 **	−0.238 **	0.048	0.043
N_VEN	0.349 **	0.272 **	0.107	0.357 **	1.000	−0.522 **	0.181 *	−0.037	0.168 *
N_VER	−0.220 **	−0.013	0.320 **	−0.549 **	−0.522 **	1.000	−0.090	−0.044	0.028 *
OUT OF BOX	0.018	0.087	−0.526 **	−0.238 **	−0.181 *	−0.090	1.000	−0.051	0.228 **
RETRIEVING	−0.096	0.020	−0.072	0.048	−0.037	−0.044	−0.051	1.000	−0.036
S/P	0.072	0.557 **	−0.187 *	0.043	0.168 *	0.028	0.228 **	−0.036	1.000

* Correlation is significant at the 0.05 level (2-tailed); ** correlation is significant at the 0.01 level (2-tailed).

## Data Availability

The data presented in this study are available on request from the corresponding author.
